# Pathogen spectrum and triple-criteria interpretation algorithm of BALF-targeted NGS in children with refractory severe community-acquired pneumonia in mountainous Northeastern Sichuan: a single-center retrospective study

**DOI:** 10.3389/fcimb.2026.1929990

**Published:** 2026-10-08

**Authors:** Rongjie Chen, Zhe Xu, Tingting He, Xiao Liu, Yi Ding, Xin Hou

**Affiliations:** 1 Department of Pediatrics, Guangyuan Central Hospital, Guangyuan, Sichuan, China; 2 Department of Pediatrics, North Sichuan Medical College Affiliated Guangyuan Central Hospital, Nanchong, Sichuan, China

**Keywords:** bronchoalveolar lavage fluid, children, coinfection, colonization discrimination, pathogen spectrum, severe community-acquired pneumonia, targeted next‑generation sequencing

## Abstract

**Background:**

Severe community-acquired pneumonia (SCAP) causes substantial pediatric hospitalization and respiratory failure in mountainous western China. Conventional microbial assays show limited sensitivity for fastidious pathogens and mixed infections. Targeted next-generation sequencing (tNGS) using bronchoalveolar lavage fluid (BALF) enables broad-spectrum pathogen detection at lower cost than whole-metagenomic sequencing. However, many previous tNGS studies directly equate multiple microbial nucleic-acid signals with true coinfection, which may lead to unnecessary combined anti-infective therapy.

**Methods:**

This single-center retrospective study enrolled 86 children aged < 14 years with refractory SCAP who underwent BALF-tNGS testing at Guangyuan Central Hospital between March 2023 and September 2024. We developed a triple-criteria interpretive algorithm combining an exploratory empirical pathogen-load cut-off (>1.0 × 10^6^ copies/mL), inflammatory biomarkers (CRP, PCT), and chest imaging to discriminate genuine active coinfection from airway colonization. This load threshold was empirical and not internally validated by ROC analysis in our dataset. Patients were stratified into four age subgroups; diagnostic concordance was assessed among 34 patients with parallel routine microbial tests.

**Results:**

In total, 229 pathogen isolates were identified. Viruses predominated (58.9%, 135/229), followed by bacteria (32.8%, 75/229). The three most frequent pathogens were Haemophilus influenzae (32.56%), Streptococcus pneumoniae (30.23%), and respiratory syncytial virus A (RSV-A, 18.60%). While 82.6% (71/86) of patients harbored ≥2 pathogen nucleic-acid signals, only 17.4% (15/86) fulfilled criteria for clinical coinfection, dominated by virus-bacterial dual infection. Viral detection rates tended to decline with increasing age, whereas bacterial positivity was higher among preschool- and school-age children. A prominent spring epidemic peak was observed during May-June 2023. Only 22.86% of paired routine microbial tests showed full concordance with tNGS. These single-center data cannot be generalized to all pediatric severe-pneumonia populations. Most abundant multi-pathogen tNGS signals represented colonization or residual dead-microbe nucleic acid instead of true active infection.

**Conclusion:**

Our triple-criteria interpretive framework offers standardized rules for grassroots mountain hospitals to reduce over-prescription of combined antibiotics and antivirals when interpreting BALF-tNGS results for children with refractory SCAP.

## Highlights

- Targeted NGS is widely applied for pediatric severe pneumonia diagnosis, yet unified clinical standards to separate viable pathogenic microbes from colonizing residual nucleic acid remain absent, especially in under-studied mountainous regions of northeastern Sichuan.
- This study established a replicable triple-index algorithm (pathogen copy load + inflammatory markers + chest radiology) to filter clinically meaningful pathogens from BALF tNGS outputs, addressing a pervasive clinical pain point of blind over-treatment after sequencing reports.
- This is the first systematic BALF tNGS epidemiological cohort focusing on children with refractory SCAP in Guangyuan hilly border prefecture, revealing distinct age-dependent pathogen shifts and seasonal spring clustering.
- All real-world data deliver localized, actionable guidance for empirical anti-infective regimens, childhood vaccination promotion, and seasonal pediatric respiratory infection prevention for county-level medical institutions in western mountainous China.
- The judgment threshold (>1.0 × 10^6^ copies/mL) requires no additional laboratory testing; however, it remains an exploratory empirical standard awaiting multi-center prospective validation.

## 1 Introduction

Severe community-acquired pneumonia (SCAP) remains the leading cause of pediatric intensive care unit (PICU) admission and acute respiratory failure among children residing in low-income and resource-limited regions worldwide, disproportionately driving pediatric critical care burden across rural, remote, and mountainous underserved settings in Asia, Africa, and Latin America (McAllister et al., 2019; Xu et al., 2021). Traditional microbial diagnostic workflows, including sputum bacterial culture and single-target PCR, have inherent limitations: poor recovery of fastidious respiratory pathogens, insufficient capacity to identify mixed infections, and lack of quantitative microbial burden evaluation, which often delays precise targeted anti-infective intervention (Dequin et al., 2026; Gutfreund et al., 2026).

Bronchoalveolar lavage fluid (BALF) targeted next-generation sequencing (tNGS) bridges the diagnostic gap between limited single-target conventional assays and expensive whole-metagenomic NGS (mNGS). Commercial tNGS panels cover over 200 common respiratory pathogens with shorter turnaround times and lower sequencing costs, making this technology accessible to prefecture-level mountain hospitals with limited medical resources (Ding et al., 2025; Qin et al., 2025). However, the ultra-high analytical sensitivity of tNGS frequently generates low-abundance microbial reads that cannot be readily discriminated from upper airway colonizers or residual non-viable microbial DNA after early antibiotic exposure (Simner et al., 2018; Chiu and Miller, 2019). Most previously published retrospective NGS studies directly counted all multi-pathogen positive signals as clinical coinfection, driving overuse of combined broad-spectrum antibiotics and antivirals, increasing medical costs and antimicrobial resistance risks (He et al., 2025; Tan et al., 2025).

Most domestic pediatric tNGS epidemiological cohorts focus on the eastern and central plain regions of China. Regional disparities in climate, vaccine coverage, and medical resources mean those published datasets cannot be directly extrapolated to hilly northeastern Sichuan. The Guangyuan hilly border prefecture features unique population mobility, a persistent humid spring climate, and incomplete coverage of childhood conjugate vaccines, and distinct regional pathogen circulation patterns, yet no published BALF tNGS-based pathogen spectrum data exist for local children with refractory SCAP.

Notably, most pediatric CAP sequencing studies rely on nasopharyngeal swabs and enroll mild-to-moderate pneumonia patients, while subjects receiving fiberoptic bronchoscopy represent treatment-refractory severe cases requiring invasive lower respiratory sampling. This subgroup is critical for optimizing sequencing interpretation standards for invasive BALF specimens, which have rarely been independently analyzed in national surveillance data. Although limited to a single-center retrospective design, our cohort uniquely characterizes the pathogen landscape of refractory severe pediatric pneumonia in northeastern Sichuan mountainous populations, filling a clear epidemiological research gap (Li et al., 2025; Lin et al., 2026).

This retrospective real-world cohort aimed to achieve four primary objectives: (i) map the full pathogen composition of BALF tNGS in local children with refractory SCAP; (ii) establish standardized triple-criteria rules to distinguish incidental multi-nucleic-acid positivity from clinically confirmed active mixed infection; (iii) describe age-stratified pathogen distribution and seasonal epidemic clustering patterns; (iv) quantify diagnostic concordance between tNGS and routine microbial testing. Our findings provide localized interpretive specifications for BALF tNGS reports and evidence-based guidance for region-specific pediatric respiratory infection prevention and anti-infective management.

## 2 Materials and methods

### 2.1 Study design, ethics, and cohort eligibility

This retrospective observational single-center study extracted electronic medical records of children aged <14 years diagnosed with SCAP admitted to the Department of Pediatrics, Guangyuan Central Hospital, from March 2023 to September 2024. SCAP diagnosis strictly complied with the 2024 Chinese Pediatric Community-Acquired Pneumonia Clinical Guidelines (Subspecialty Group of Respiratory, the Society of Pediatrics, Chinese Medical Association et al., 2024a).

#### 2.1.1 Inclusion criteria

1. age <14 years;
2. definite clinical diagnosis of severe community-acquired pneumonia;
3. fiberoptic bronchoscopy and BALF collection for tNGS testing completed during hospitalization.

#### 2.1.2 Exclusion criteria

1. non-infectious pulmonary lesions;
2. incomplete clinical laboratory, imaging, or sequencing records;
3. systemic broad-spectrum antibiotics administered within 72 h prior to BAL sampling.

Note: Exclusion of patients receiving recent broad-spectrum antibiotics may introduce selection bias and limits population representativeness; this point is further elaborated in the limitation section.

The final eligible cohort included 86 children. All subjects were divided into four standard pediatric respiratory epidemiological age subgroups: 0-12 months (infants), 13-36 months (toddlers), 37-60 months (preschoolers), and >60 months (school-age children).

All bronchoscopy-lavage procedures were routine clinical management for refractory pneumonia and were not performed solely for research purposes. Ethical approval was granted (No. GYZXLL2023017). Given the de-identified retrospective data analysis, the informed consent requirement was waived by the ethics committee. The study was conducted in accordance with the Declaration of Helsinki.

### 2.2 BALF sampling and tNGS laboratory protocol

All bronchoscopy and bronchoalveolar lavage procedures were performed by attending pediatric respiratory physicians following national pediatric BAL operation guidelines (2024) (Yongjun et al., 2024). After topical lidocaine mucosal anesthesia, 1-2 mL/kg sterile normal saline was instilled into lesion-affected lung lobes. Only specimens with a fluid recovery rate ≥30 % were submitted for sequencing analysis.

The commercial PathoSeq^®^ targeted respiratory pathogen panel (CapitalBio Technology) was used, covering >80 viral pathogens and >100 bacterial/atypical respiratory microbes plus partial fungal targets. Nucleic acid extraction, library construction, and sequencing were performed fully in accordance with manufacturer standardized protocols. Bioinformatic pipelines included host-genome read removal, quality filtering, and background-contamination subtraction using blank negative-control libraries to generate pathogen copy concentrations (copies/mL) for each detected microbe.

We adopted an exploratory empirical cut-off of >1.0 × 10^6^ copies/mL to define high-concentration pathogens, which has been utilized in multiple pediatric BALF-tNGS retrospective cohorts both in China and internationally (Jabeen et al., 2022; Wang et al., 2024; Haiqin et al., 2024; Jing et al., 2026; Liang et al., 2026). This threshold has not been prospectively validated by ROC curve within our own dataset and is not an official diagnostic guideline gold standard. Low-concentration positive signals without corresponding clinical infection manifestations were classified as airway colonization or specimen background contamination. For example, low-copy reads of *Aspergillus* or *Mycobacterium tuberculosis* without compatible pulmonary cavity/nodule changes on chest CT and without elevated inflammatory markers were judged as colonization or contamination, whereas high-copy pathogen signals together with matching clinical-radiological findings supported clinically relevant infection. Sequencing depth and raw read filtering parameters were standardized across all BALF samples to eliminate detection bias.

### 2.3 Synchronous conventional microbial testing

Thirty-four of the 86 enrolled patients completed routine microbial examinations within 24 h of BAL sampling, including sputum/BAL bacterial culture, Gram staining, and multi-target respiratory PCR panels.

Note: This comparative subset represents a convenience clinical sample, not pre-defined by study protocol; patients with more severe clinical manifestations were more likely to receive these parallel tests, which brings potential selection bias.

Test concordance was categorized into three tiers for comparative statistical analysis: fully consistent, partially consistent, and totally-inconsistent. Brief clinical vignettes for four totally-inconsistent cases are provided in **Supplementary Text S1**.

### 2.4 Clinical data extraction and definition of clinical mixed infection

Extracted clinical variables included gender, age (converted to months), BAL sampling date, fever/wheezing clinical manifestations, peripheral white blood cell count, neutrophil percentage, CRP, PCT, chest CT/DR imaging results, and full tNGS pathogen copy reports.

Definition of clinically confirmed active mixed infection: ≥2 pathogens detected at high load (1.0 × 10^6^ copies/mL), plus at least one supportive clinical infection marker: persistent fever, pulmonary consolidation on chest imaging, CRP > 10 mg/L, OR PCT > 0.5 ng/mL. Cases carrying multiple low-copy pathogen signals without matching inflammatory or radiological evidence were only recorded as multi-pathogen nucleic-acid positivity and excluded from clinical coinfection statistics (Wang et al., 2026).

Important: Inflammatory biomarkers are not a mandatory requirement; pulmonary imaging or persistent fever alone can support an active coinfection diagnosis.

Peripheral blood high-sensitivity C-reactive protein (hs-CRP) was measured via latex-enhanced immunonephelometry on a CRP-M100 analyzer (Mindray Medical, Shenzhen, China), using the commercially available high-sensitivity C-reactive protein assay kits from the same manufacturer. Whole-blood specimens collected in EDTA-K3 anticoagulant tubes were used for hs-CRP measurement. Procalcitonin (PCT) was quantified by immunofluorescence assay with procalcitonin assay kits (Sichuan Orienter Biotechnology Co., Ltd., Sichuan, China). Serum samples were collected in separator-gel procoagulant tubes without anticoagulants for PCT testing. All biomarker measurements were performed on clinical laboratory automatic analyzers following the manufacturer’s standard operating procedures. Cut-off values CRP > 10 mg/L and PCT > 0.5 ng/mL were adopted according to Chinese pediatric severe community-acquired pneumonia clinical guidelines (2024).

### 2.5 Statistical analysis

All statistical analyses were performed using IBM SPSS 26.0 and Python 3.9. Categorical variables were reported as case counts and proportions with binomial exact 95% confidence intervals (CIs). Inter-group detection rate comparisons adopted chi-square tests; Fisher’s exact test was applied when theoretical cell frequencies were <5. Two-tailed P < 0.05 indicated descriptive statistical tendency.

This exploratory descriptive study lacked pre-specified sample-size power calculation. Multiple inter-group comparisons were conducted without Bonferroni-type multiplicity correction. Consequently, obtained P-values serve only for exploratory descriptive purposes and may inflate type-I error risk; they should not be over-interpreted as definitive inter-group causal evidence.

Graphical visualizations were generated via GraphPad Prism 9 and Matplotlib.

**Supplementary Materials** legend: **Supplementary Table S1**: Baseline inflammatory indicators, imaging manifestations, and stratified clinical data of all 86 patients; **Supplementary Table S2**: Profiles of 15 clinically confirmed active mixed-infection cases; **Supplementary Figure S1**: Monthly case-distribution plot of enrolled patients; **Supplementary Text S1**: Brief clinical vignettes for four totally-inconsistent tNGS-routine-test cases. All shared datasets are fully de-identified with zero patient-identifiable information.

All fully de-identified raw aggregated clinical and sequencing data supporting this study are provided in clearly-labeled supplementary datasets for peer review (see **Supplementary Materials** legend).

## 3 Results

### 3.1 Baseline demographic characteristics

The cohort contained 43 female (50.0%) and 43 male (50.0%) children, with a mean age of 45.2 ± 48.3 months (range, 1-147 months). Age subgroup distribution: 0-12 months (27 patients, 31.4%), 13-36 months (26, 30.2%), 37-60 months (17, 19.8%), >60 months (16, 18.6%). All patients presented fever and cough; wheezing symptoms were only observed in infants and toddlers aged <36 months, with no wheezing cases in preschool and school-age children. No subjects received broad-spectrum antibiotics within 72 h before BAL sampling. Baseline inflammatory indicators, imaging manifestations, and stratified clinical data of all 86 patients are summarized in **Supplementary Table S1**.

### 3.2 Overall pathogen composition and top 10 detected microbes

A total of 229 independent pathogen isolates were recovered from all 86 BALF samples. Viral strains accounted for 58.9% (135/229), bacteria 32.8% (75/229), and atypical bacteria and fungi 8.3% (19/229). The top 10 most prevalent pathogens by positive case count are listed in **Table 1**.

**Table 1:** Top 10 pathogens detected via BALF tNGS in 86 SCAP children.

Rank	Pathogen	Positive cases	Detection rate (%)
1	*Haemophilus influenzae*	28	32.56
2	*Streptococcus pneumoniae*	26	30.23
3	Respiratory syncytial virus A (RSVA)	16	18.60
4	Rhinovirus A	13	15.12
5	Parainfluenza virus 3	12	13.95
6	*Mycoplasma pneumoniae*	12	13.95
7	Human bocavirus 1	11	12.79
8	Coronavirus OC43	9	10.47
9	Adenovirus C	8	9.30
10	*Moraxella catarrhalis*	7	8.14

Detection rate = positive patients/total n = 86.

### 3.3 Multi-pathogen nucleic-acid positivity *vs.* clinically verified mixed infection

Seventy-one patients (82.6%) displayed ≥2 distinct pathogen nucleic-acid signals on tNGS. After screening by the triple-criteria system, only 15 patients (17.4%) met diagnostic standards for authentic active mixed infection. The remaining 56 multi-positive cases were classified as airway colonization or residual microbial nucleic acid without evidence of active infection.

Among the 15 clinically confirmed coinfection cases, virus-bacterial combination predominated (8/15, 53.3%), followed by virus-virus coinfection (4/15, 26.7%) and bacteria-bacteria coinfection (3/15, 20.0%) (**Supplementary Table S2**).

### 3.4 Age-stratified pathogen detection differences

Viral detection rates tended to decrease with advancing age, whereas bacterial positivity appeared higher in preschool and school-age subgroups. Multi-nucleic-acid positivity rates peaked in infants and toddlers (92.6% and 92.3%) and dropped among school-age children (50.0%). Observed inter-group tendencies should be interpreted cautiously given uncorrected multiple comparisons (**Table 2**).

**Table 2:** Age-stratified pathogen detection rates with binomial exact 95% CIs.

Age group (months)	n	Virus positive n (%) [95% CI]	Bacteria positive n (%) [95% CI]	Multi-nucleic acid positive n (%) [95% CI]	P value
0-12	27	20 (74.1) [53.7-88.9]	15 (55.6) [35.3-74.5]	25 (92.6) [75.7-99.1]	<0.05
13-36	26	22 (84.6) [65.1-95.6]	18 (69.2) [48.2-85.7]	24 (92.3) [74.9-99.1]	<0.05
37-60	17	14 (82.4) [56.6-96.2]	16 (94.1) [71.3-99.9]	14 (82.4) [56.6-96.2]	<0.05
>60	16	7 (43.8) [19.8-69.5]	12 (75.0) [47.6-92.7]	8 (50.0) [24.7-75.3]	<0.05
Total	86	63 (73.3) [62.8-82.0]	61 (70.9) [60.4-80.0]	71 (82.6) [72.9-89.6]	—

95% binomial exact confidence intervals are presented for proportions. P-values derived from multiple pairwise comparisons without multiplicity correction and serve for exploratory description only, should not be interpreted as definitive group differences.

### 3.5 High-load pathogen distribution

Ninety-nine of 229 total pathogen isolates (43.2%) exceeded the exploratory empirical cut-off >1.0 × 10^6^ copies/mL. Viruses accounted for 67.7% (67/99) of all high-load pathogens; RSV-A and *Mycoplasma pneumoniae* were the two most frequent high-burden microbes associated with clinical disease. All low-load signals were dominated by coagulase-negative staphylococci, uniformly classified as colonization without clinical pathogenicity.

### 3.6 Seasonal epidemic distribution

A prominent seasonal outbreak peak occurred during May-June 2023, enrolling 43 patients, accounting for exactly 50% of the entire cohort. A minor secondary case peak appeared in August 2024; all other months showed scattered low admission volumes for refractory SCAP requiring bronchoscopy and tNGS testing. Monthly case distribution is visualized in **Supplementary Figure S1**.

### 3.7 Diagnostic concordance between tNGS and conventional microbial assays

Among 34 paired samples with simultaneous routine microbial testing, only 8 cases (22.86%) achieved fully consistent pathogen results. Twenty-two cases (62.86%) showed partial consistency: conventional tests captured partial pathogens, while tNGS identified additional low-abundance fastidious microbes missed by culture or single-target PCR. Four cases (11.43%) were totally inconsistent, with negative routine test results but clinically relevant high-load pathogens detected by tNGS matching chest imaging and elevated inflammatory markers (**Table 3**).

**Table 3:** Concordance classification of tNGS versus synchronous conventional microbial tests (n = 34).

Concordance category	Case number	Proportion (%)	Definition
Fully consistent	8	22.86	Identical pathogens detected by two detection platforms
Partially consistent	22	62.86	Conventional tests detected partial pathogens; tNGS identified extra low-abundance microbes
Totally inconsistent	4	11.43	Conventional results negative; tNGS high-load pathogens consistent with clinical pneumonia manifestations

Brief clinical vignettes for four totally inconsistent cases are available in **Supplementary Text S1**.

## 4 Discussion

### 4.1 Core regional microbiological and clinical findings

This is the first systematic BALF tNGS epidemiological study focusing on children with refractory SCAP in the hilly border city of Guangyuan, western Sichuan. Our cohort confirms viral predominance in pediatric severe lower respiratory infection, consistent with multinational PERCH global pediatric pneumonia surveillance data (Feng et al., 2023; GBD 2023 Lower Respiratory Infections and Antimicrobial Resistance Collaborators, 2026). Locally, RSV-A ranked as the most prevalent viral pathogen, matching the annual spring surge of infant wheezing pneumonia observed in routine clinical practice at our center (Chang et al., 2024; Chen et al., 2026).

The most critical methodological advancement of this study lies in the triple-factor stratification algorithm separating colonization signals from true mixed infection, resolving a pervasive flaw in prior NGS retrospective research that severely overestimated clinical coinfection prevalence. The raw 82.6% multi-pathogen nucleic-acid positivity rate cannot independently guide clinical anti-infective decision-making; only 17.4% of patients satisfied strict combined microbial burden, inflammatory, and radiological criteria for authentic coinfection, with virus-bacterial dual infection as the dominant subtype. Without multi-index filtering, grassroots clinicians in mountain hospitals frequently prescribe blind combined broad-spectrum antibiotics and antivirals, accelerating local antimicrobial resistance (Simner et al., 2018).

Age-dependent pathogen tendencies closely correspond to pediatric immune maturation trajectories. Immature cellular immunity in infants <12 months leads to higher viral exposure and elevated multi-pathogen sequencing signals (Kollmann et al., 2017; Lloyd and Saglani, 2023). Older preschool and school-age children enter group childcare environments, increasing carriage and invasive infection risk of vaccine-preventable *Haemophilus influenzae* and *Streptococcus pneumoniae* (Huang et al., 2005; Du et al., 2024). The high local detection rates of these two bacteria reflect incomplete Hib and pneumococcal conjugate vaccine coverage in surrounding rural mountain townships, highlighting urgent demand for rural vaccination outreach programs (Lai et al., 2022; Li et al., 2024). Most prior pediatric tNGS publications rely purely on copy-number cut-offs to define coinfection without integrating imaging and inflammatory markers; our triple-criteria workflow mitigates over-diagnosis risk, yet limitations remain: the empirical load threshold lacks multi-center prospective validation, and inflammatory biomarkers can also increase due to non-infectious conditions.

### 4.2 External applicability of the triple-criteria interpretation algorithm

The triple-index judgment framework combining pathogen copy load, inflammatory biomarkers, and chest imaging proposed in this study possesses strong external promotion value for western mountain grassroots medical institutions. Most county-level hospitals lack uniform operational standards to distinguish colonization nucleic acid from active infection after tNGS testing, resulting in widespread over-medication. The >1.0 × 10^6^ copies/mL load threshold adopted in our research aligns with domestic pediatric respiratory sequencing consensus, requiring no extra laboratory testing costs and suitable for direct popularization in resource-limited prefectural and county hospitals across western mountainous China (Haiqin et al., 2024). Subsequent multi-center prospective external validation of this judgment standard is worthy of further independent research to expand its generalizability nationwide.

### 4.3 Comparison with domestic and international pediatric NGS cohorts

The top pathogen spectrum (RSV-A, *Haemophilus influenzae*, *Streptococcus pneumoniae*) aligns with national Chinese pediatric CAP multi-center surveillance datasets (Huang et al., 2005; Du et al., 2024; Li et al., 2024). However, our raw multi-pathogen positivity rate far exceeds cohorts relying solely on culture or single-target PCR, attributable to the superior analytical sensitivity of tNGS for low-abundance fastidious organisms (Lai et al., 2022; Li et al., 2024). Large-scale U.S. pediatric pneumonia cohorts reported clinical coinfection rates up to 45.3%, substantially higher than our 17.4% verified mixed infection proportion. This discrepancy arises from two key methodological and population differences: foreign studies did not integrate quantitative pathogen load thresholds to exclude colonization signals, and their cohorts included mild-to-moderate CAP patients, whereas our study exclusively enrolled refractory SCAP requiring invasive bronchoscopy sampling.

Two recent real-world pediatric tNGS studies published in Frontiers journals also reported variable coinfection rates (Yan et al., 2026; Zhu et al., 2026). Yan et al. emphasized that tNGS results should be interpreted in combination with clinical phenotypes rather than relying purely on sequencing outputs (Yan et al., 2026). In a PICU-based severe pediatric pneumonia cohort, Zhu et al. further warned that high multi-pathogen positivity from tNGS alone cannot be equated to authentic active infection (Zhu et al., 2026). Such heterogeneity across published cohorts is largely driven by differences in patient inclusion criteria and pathogen-interpretation strategies instead of true epidemiological divergence. These observations further underline the necessity of local real-world sequencing data to formulate region-specific empirical treatment guidelines for mountain-area pediatric populations.

### 4.4 Study strengths

Fills an epidemiological blank in under-studied hilly western Sichuan, utilizing lower respiratory BALF instead of nasopharyngeal swabs to reflect lesion-site true pathogens rather than upper airway colonizers.

Establishes a standardized triple-criterion framework to distinguish viable pathogenic microbes from residual colonizing nucleic acid, providing replicable low-cost interpretive standards for grassroots prefecture-level hospitals.

Incorporates paired routine microbial testing to quantitatively evaluate the incremental diagnostic value of tNGS relative to traditional culture and PCR workflows.

Simultaneously analyzes age stratification and seasonal epidemic clustering to generate actionable pediatric respiratory infection prevention and vaccination strategies for local public health practice.

Of note, the pathogen load threshold of >1.0×10^6^ copies/mL was an empirical exploratory cut-off adopted from retrospective BALF-based pediatric pneumonia cohorts in China, which stratified high-versus-low pathogen load for *Mycoplasma pneumoniae*; no universal tNGS quantitative cut-offs for discriminating coinfection from colonization are currently available, and this threshold was not optimized via ROC within our present dataset.

### 4.5 Study limitations

This study has several inherent limitations that must be fully acknowledged.

1. Single-center retrospective design brings obvious selection bias: only children with refractory severe SCAP who underwent fiber-optic bronchoscopy were enrolled, so the results cannot be extrapolated to mild community-acquired pneumonia in children. Exclusion of patients receiving broad-spectrum antibiotics within 72 h before BAL further restricts the study population.
2. The total sample size is relatively small and age subgroups are unbalanced, reducing statistical power for inter-subgroup comparisons; no pre-specified sample-size calculation and lack of multiple testing correction may raise type I error risk.
3. Only 34 patients completed paired conventional microbial testing. This subgroup represents convenience sampling driven by clinical decision-making rather than study protocol; selection bias exists, and concordance analysis cannot fully validate overall tNGS diagnostic performance against the culture gold standard.
4. tNGS technology cannot completely distinguish viable pathogens from free residual microbial nucleic acid in specimens (Lai et al., 2022); even after multi-index screening, minor misclassification of low-level colonization cannot be fully eliminated. In addition, CRP and PCT have limited discriminative value for virus-dominant infection, so the triple-criteria algorithm relies on comprehensive clinical-imaging evidence instead of biomarkers alone.
5. Complete long-term follow-up outcome data (mechanical ventilation duration, hospital length of stay, standardized disease severity score) were not systematically archived in electronic medical records, prohibiting correlation analysis between pathogen subtypes and disease prognosis.

### 4.6 Local clinical and public health implications

Age-stratified empirical anti-infective regimens: Infants admitted during spring epidemic peaks with severe wheezing pneumonia should prioritize anti-RSV supportive and targeted therapy. School-age children presenting with large pulmonary consolidation require initial empirical coverage of *Haemophilus influenzae* and *Streptococcus pneumoniae.*

Standardized local tNGS report interpretation rules: Hospitals in western mountain regions should adopt our triple-index standard (pathogen copy load + CRP/PCT + chest imaging) to avoid over-prescription of combined antibiotics and antivirals.

Vaccination promotion: Strengthen publicity and accessibility of Hib and pneumococcal conjugate vaccines for rural mountain families to reduce the burden of vaccine-preventable bacterial pneumonia. Guangyuan is a hilly border prefecture with humid spring climate that contributes to obvious spring respiratory virus outbreaks. Uneven vaccine coverage in surrounding rural townships partly explains high detection rates of *Haemophilus influenzae* and *Streptococcus pneumoniae* observed in this cohort.

Seasonal targeted prevention: Enforce infant respiratory isolation, standardized hand-hygiene education, and outpatient screening every May-June during the local RSV epidemic peak to lower pediatric hospitalization volume.

### 4.7 Concluding remarks

Viral pathogens dominate BALF tNGS-positive results in children with refractory severe community-acquired pneumonia from mountainous Guangyuan, western Sichuan, with RSV-A, *Haemophilus influenzae*, and *Streptococcus pneumoniae* as the three most prevalent microbes. The high raw multi-pathogen nucleic-acid positivity rate largely represents airway colonization or residual dead microbial DNA rather than clinically relevant active mixed infection. Pathogen distribution exhibits clear age differentiation and spring seasonal clustering. This single-center descriptive cohort provides preliminary regional epidemiological evidence to standardize BALF tNGS report interpretation and guide empirical anti-infective management, and should not be broadly extrapolated to other pediatric populations. Multi-center prospective cohorts integrating complete clinical outcome endpoints are required to validate generalizable standardized treatment guidance for northeastern Sichuan mountain pediatric populations.

## Data Availability

The original contributions presented in the study are included in the article/**Supplementary Material**. Further inquiries can be directed to the corresponding author.
