# Supplementary material for: Pathogen spectrum and triple-criteria interpretation algorithm of BALF-targeted NGS in children with refractory severe community-acquired pneumonia in mountainous Northeastern Sichuan: a single-center retrospective study
Source: Front Cell Infect Microbiol. 2026 Oct 8;16:1929990. doi: 10.3389/fcimb.2026.1929990 (PMC13686821; doi:10.3389/fcimb.2026.1929990)
Supplement: Supplementary file 1 [file Supplementaryfile1.docx]

Supplementary Text: Brief clinical descriptions for four totally‑inconsistent cases presented in Table 3

Case 1

Patient characteristics: 2‑year‑6‑month‑old female

Admission diagnosis: Severe *Mycoplasma pneumoniae* pneumonia, tracheobronchitis

High‑load pathogens detected by tNGS: *Mycoplasma pneumoniae* (49721 reads, >1.0 × 10⁶ copies/mL), with macrolide‑resistant 23S rRNA gene mutation detected

Synchronous conventional microbial results: Positive for Gram‑positive cocci on BALF smear; negative for sputum culture, acid‑fast staining and fungal smear

Core inconsistency: tNGS identified high‑load *Mycoplasma pneumoniae* as the dominant pathogen, while conventional tests only detected non‑specific Gram‑positive cocci with no evidence of mycoplasma infection, showing no overlapping clinically relevant pathogens.

Case 2

Patient characteristics: 2‑month‑4‑day‑old male

Admission diagnosis: Severe pneumonia, respiratory failure, myocardial injury, respiratory syncytial virus (RSV) infection

High‑load pathogens detected by tNGS: Human respiratory syncytial virus A (RSV‑A, 41577 reads, >1.0 × 10⁶ copies/mL), cytomegalovirus (CMV, 30875 reads, >1.0 × 10⁶ copies/mL)

Synchronous conventional microbial results: Positive for *Pseudomonas aeruginosa* on sputum culture; no positive viral detection results

Core inconsistency: tNGS revealed a dominant high‑load viral infection pattern, while conventional culture only detected *Pseudomonas aeruginosa* with no positive viral findings, resulting in completely discordant pathogen identification.

Case 3

Patient characteristics: 3‑month‑12‑day‑old female

Admission diagnosis: Bronchopneumonia

High‑load pathogens detected by tNGS: Human respiratory syncytial virus A (RSV‑A, 22779 reads, >1.0 × 10⁶ copies/mL)

Synchronous conventional microbial results: Positive for adenovirus and severe acute respiratory syndrome coronavirus 2 (SARS‑CoV‑2) on respiratory virus panel PCR; no positive RSV detection

Core inconsistency: tNGS identified high‑load RSV‑A as the main pathogen, while conventional PCR only detected adenovirus and SARS‑CoV‑2 with no RSV‑A positivity, showing complete mismatch in viral pathogen detection.

Case 4

Patient characteristics: 9‑year‑11‑month‑old female

Admission diagnosis: *Mycoplasma pneumoniae* pneumonia, rhinovirus infection

tNGS detection results: Only low‑load herpes simplex virus 1 (HSV‑1, 125 reads, <1.0 × 10³ copies/mL), no high‑load signals of rhinovirus or *Mycoplasma pneumoniae*.

Synchronous conventional microbial results: Positive for human rhinovirus on respiratory virus panel PCR; no other positive pathogens

Core inconsistency: Clinical diagnosis and conventional tests confirmed rhinovirus infection, while tNGS failed to detect the target rhinovirus pathogen and only identified irrelevant low‑load HSV‑1, with no consistent pathogen findings between the two methods.

Supplementary Figure S1

Monthly case distribution of children with refractory severe SCAP (Mar 2023–Sep 2024)

Monthly patient count

| Sampling month | Cases |
| --- | --- |
| 2023/3 | 6 |
| 2023/4 | 7 |
| 2023/5 | 22 |
| 2023/6 | 21 |
| 2023/7 | 4 |
| 2023/8 | 3 |
| 2023/9 | 2 |
| 2023/10 | 2 |
| 2023/11 | 3 |
| 2023/12 | 0 |
| 2024/1 | 0 |
| 2024/2 | 0 |
| 2024/3 | 1 |
| 2024/4 | 1 |
| 2024/5 | 1 |
| 2024/6 | 2 |
| 2024/7 | 2 |
| 2024/8 | 5 |
| 2024/9 | 4 |

Figure legend: Monthly distribution of children with refractory severe community‑acquired pneumonia (SCAP) receiving BALF targeted NGS testing from March 2023 to September 2024. The x‑axis represents sampling months, and the y‑axis denotes monthly patient counts. A major epidemic peak occurred in May‑June 2023, with a total of 43 patients accounting for 50% of all enrolled subjects; a minor secondary case peak was observed in August 2024. Patient admissions remained low and scattered in all other months.

**Supplementary Materials**

Supplementary Table S1: Baseline clinical, inflammatory, and imaging data of all 86 enrolled children

| Characteristic | Overall cohort (n=86) | 0–12 months (n=27) | 13–36 months (n=26) | 37–60 months (n=17) | >60 months (n=16) |
| --- | --- | --- | --- | --- | --- |
| **Gender, n (%)** |  |  |  |  |  |
| Male | 43 (50.0) | 14 (51.9) | 13 (50.0) | 8 (47.1) | 8 (50.0) |
| Female | 43 (50.0) | 13 (48.1) | 13 (50.0) | 9 (52.9) | 8 (50.0) |
| **Age, months** |  |  |  |  |  |
| Mean ± SD | 45.2 ± 48.3 | 5.8 ± 3.5 | 22.1 ± 7.2 | 48.5 ± 6.8 | 102.4 ± 28.7 |
| Range | 1–147 | 1–12 | 13–36 | 37–60 | 61–147 |
| **Clinical symptoms, n (%)** |  |  |  |  |  |
| Fever | 86 (100.0) | 27 (100.0) | 26 (100.0) | 17 (100.0) | 16 (100.0) |
| Cough | 86 (100.0) | 27 (100.0) | 26 (100.0) | 17 (100.0) | 16 (100.0) |
| Wheezing | 16 (18.6) | 9 (33.3) | 7 (26.9) | 0 (0.0) | 0 (0.0) |
| **Chest imaging, n (%)** |  |  |  |  |  |
| Imaging type |  |  |  |  |  |
| DR | 43 (50.0) | 14 (51.9) | 13 (50.0) | 8 (47.1) | 8 (50.0) |
| CT | 43 (50.0) | 13 (48.1) | 13 (50.0) | 9 (52.9) | 8 (50.0) |
| Imaging manifestation |  |  |  |  |  |
| Patchy infiltration | 58 (67.4) | 18 (66.7) | 18 (69.2) | 11 (64.7) | 11 (68.8) |
| Pulmonary consolidation | 28 (32.6) | 9 (33.3) | 8 (30.8) | 6 (35.3) | 5 (31.2) |
| **Inflammatory biomarkers, mean ± SD** |  |  |  |  |  |
| WBC (×10⁹/L) | 13.6 ± 4.2 | 13.2 ± 3.8 | 13.5 ± 4.1 | 13.8 ± 4.5 | 14.2 ± 4.8 |
| Neutrophil percentage (%) | 65.2 ± 8.3 | 62.5 ± 7.8 | 64.8 ± 8.1 | 67.2 ± 8.5 | 69.5 ± 8.8 |
| CRP (mg/L) | 32.1 ± 12.4 | 23.7 ± 11.2 | 28.9 ± 11.8 | 36.6 ± 12.1 | 44.5 ± 12.8 |
| PCT (ng/mL) | 1.0 ± 0.4 | 0.8 ± 0.3 | 0.97 ± 0.35 | 1.11 ± 0.38 | 1.25 ± 0.42 |
| **Pre-BAL antibiotics, n (%)** |  |  |  |  |  |
| No broad-spectrum antibiotics within 72h | 86 (100.0) | 27 (100.0) | 26 (100.0) | 17 (100.0) | 16 (100.0) |

Table note: All data are presented as n (%) for categorical variables and mean ± standard deviation (SD) for continuous variables. No patients received broad-spectrum antibiotics within 72 hours before bronchoalveolar lavage (BAL) sampling, meeting the study inclusion criteria.

Supplementary Table S2: Subtypes of clinically verified active mixed infection among 15 patients

| Mixed infection pattern | Number of cases | Constituent ratio (%) |
| --- | --- | --- |
| Virus + Bacteria | 8 | 53.33 |
| Virus + Virus | 4 | 26.67 |
| Bacteria + Bacteria | 3 | 20.00 |
